# 91-month follow-up of solitary punctate chorioretinitis in a Chinese patient

**DOI:** 10.1186/s12886-024-03576-6

**Published:** 2024-07-19

**Authors:** Chu Liu, Mengke Liu, Xinyue Lan, Junjie Zhu, Zhengwei Zhang

**Affiliations:** 1 Department of Ophthalmology, Jiangnan University Medical Center, 68 Zhongshan Road, Wuxi, 214002 China; 2 Department of Ophthalmology, Wuxi No.2 People’s Hospital, Affiliated Wuxi Clinical College of Nantong University, Wuxi, China

**Keywords:** Solitary punctate chorioretinitis (SPC), Punctate inner choroidopathy (PIC), Long-term follow-up, Quiescent choroidal neovascularization (CNV), Optical coherence tomography (OCT)

## Abstract

**Background:**

Solitary Punctate Chorioretinitis (SPC) is a recently identified form of punctate inner choroidopathy (PIC) characterized by a single lesion in the fovea of the macula. Previous studies with a maximum follow-up of 48 months were insufficient. Our review uncovered a case sustained for 91 months.

**Case presentation:**

A 28-year-old young woman experienced with sudden visual loss in her right eye. Comprehensive examinations, including assessment of best-corrected visual acuity (BCVA), slit-lamp biomicroscopy, noncontact tonometry, fundus fluorescein angiography (FFA), fundus autofluorescence (FAF), optical coherence tomography angiography (OCTA), perimetry, and microperimetry, were conducted. Over 91 months, the lesion slightly enlarged, remained yellow-white and punctate, and stayed in the central macula of the posterior pole. OCT images depicted subsidence in the inner nuclear layer (INL), the outer plexiform layer (OPL), photoreceptor layer, and disruption of the external limiting membrane (ELM), ellipsoid zone, and retinal pigment epithelium (RPE)/Bruch’s membrane complex. Retinal herniation, focal choroidal excavation (FCE), and abnormal vessels in the choriocapillaris were noted. At the slab of the choriocapillaris, OCTA demonstrated that the lesion resembled a linear vascular structure, distinct from the structure of normal choriocapillaris. This confirmed the lesion as an abnormal vascular formation. FAF revealed a punctate hypo-autofluorescence lesion and abnormal hyper-autofluorescence near the optic disc and macula. FFA demonstrated a punctate hyper-fluorescent lesion inferotemporal to the fovea. The vascular structure remained stable without fluid exudation on OCT images, hence anti-vascular endothelial growth factor (anti-VEGF) treatment was not administered. Visual acuity improved from counting fingers to 0.07 in 52 days, reached 0.6 after 15 months, remained at 0.6 from 56 to 80 months, and returned to 0.8 after 91 months, although accompanied by local scotomas. The lesion pattern slightly enlarged without scarring.

**Conclusions:**

Throughout long-term follow-up, we had long suspected the presence of choroidal neovascularization (CNV) and found the FCE in the last visit. Eventually, we concluded that SPC could potentially constitute a distinct subtype of PIC. The patient received no treatment, and vision recovered to 0.8. If CNV is suspected in SPC, anti-VEGF treatment may not be necessary without activity on OCT, but close monitoring is essential.

## Background

Punctate inner choroidopathy (PIC) was initially described by Watzke and colleagues in 1984. The precise cause of PIC remains enigmatic, and its underlying mechanisms remain inadequately understood. Like other inflammatory conditions grouped under the “white dot syndromes” category, PIC is believed to have an autoimmune basis, developing within a framework of polygenic susceptibility, potentially triggered by environmental factors such as infections, immunizations, or stress [1, 2]. It predominantly afflicts young, myopic females and is characterized by common clinical manifestations, including diminished visual acuity, photopsia (perceived flashes of light), and central scotoma (a blind spot in central vision). Ocular examination typically reveals multiple deep lesions ranging from 100 μm to 300 μm in size, manifesting as yellow-white spots primarily located in the posterior pole of the eye [3, 4]. Over time, these spots progress into atrophic scars. Vision loss is typically attributed to either the emergence of new lesions observable during fundus examination or the occurrence of choroidal neovascularization (CNV) [5–8].

Notably, researchers have recently reported a distinct subtype of PIC known as solitary punctate chorioretinitis (SPC) [9]. SPC shares typical features with PIC, such as the presence of yellow-white lesions, which appear hyper-fluorescent on fundus fluorescein angiography (FFA). SPC is distinguished by its exclusive presentation as a solitary lesion located in the macula [9]. However, their follow-up period was deemed insufficient. In our recent retrospective study, we documented a case of SPC with an extended 91-month follow-up period.

## Case description

**Fig. 1:**
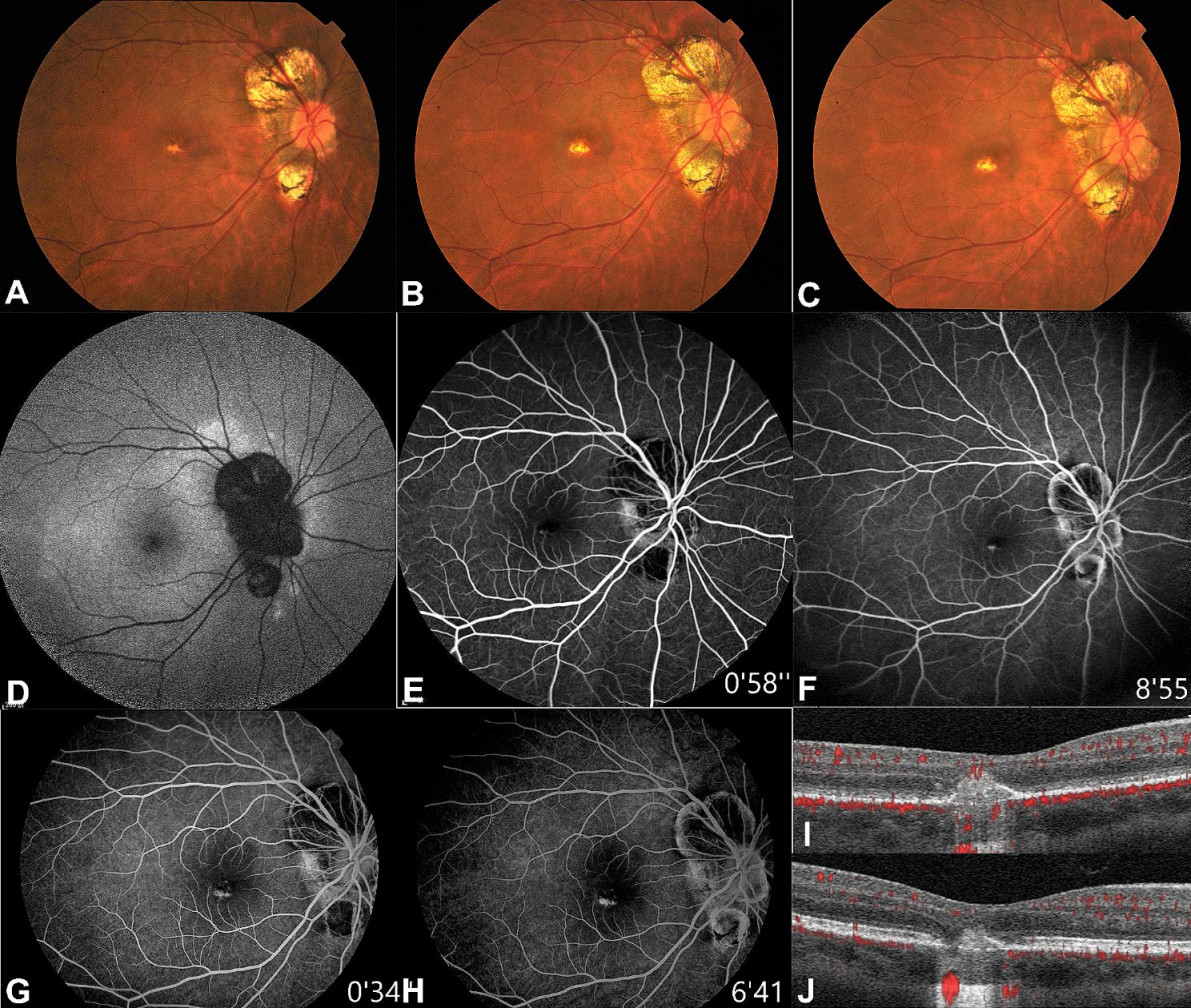
At the first visit, the fundus photograph showed a yellow-white lesion in the macular area (**A**). Over the next fifty-six months (**B**) or seventy months (**C**), the lesion enlarged slightly. Twelve days after the initial diagnosis, the FAF image showed a punctate hypo-autofluorescence lesion and abnormally hyper-autofluorescence in the posterior (**D**). Early-phase FFA revealed a hyper-fluorescent lesion in the macula (**E**). Late-phase FFA showed a small dot lesion that was stained (**F**). Three months later, early-phase (**G**) and late-phase (**H**) FFA showed that the lesion still had staining. Twelve days after the initial visit, OCT showed a “hump-like” moderately hyper-reflective nodule in the outer retina (**I**). three months after the initial visit, the height of the moderately hyper-reflective nodule decreased (**J**)

A 28-year-old Chinese female, with a bilateral refractive error of -6D myopia, presented with a ten-day history of visual impairment in her right eye. On ophthalmic examination, visual acuity in the right eye was hand motion close to the face, while the left eye had a visual acuity of 1.0. The patient’s visual acuity remained at 0.6 from 56 to 80 months, and returned to 0.8 after 91 months. A comprehensive ophthalmic evaluation was performed. At the first visit, fundus photography revealed a solitary yellow-white lesion in the central macula of the posterior pole (Fig. 1A), which slightly enlarged after fifty-six months (Fig. 1B) and seventy months (Fig. 1C). Twelve days after the initial diagnosis, fundus autofluorescence (FAF) revealed abnormally hyper-autofluorescence in the vicinity of the optic disc and macula, with the lesion appearing fused to the normally hypo-autofluorescence in the macular fovea, rendering it challenging to distinguish (Fig. 1D). At the same time, in the early phase of FFA, hyper-fluorescence was observed at 58 s in the case, suggesting the location of the lesion (Fig. 1E). In the late phase of FFA, the lesion was hyper-fluorescent with staining at 8 min and 55 s (Fig. 1F). Three months later, early-phase FFA at 34 s (Fig. 1G) and late-phase FFA at 6 min and 41 s (Fig. 1H) showed that the punctate lesion and staining were slightly enlarged. At initial diagnosis, optical coherence tomography (OCT) showed a “hump-like” moderately hyper-reflective nodule in the outer retina (Fig. 1I). After three months, the moderately hyper-reflective nodule on OCT was still present, but at a lower height indicating gradual recovery of the lesion (Fig. 1J). The fundus photograph and FFA showed no abnormalities in the left eye within three months (Figure S1) and at the last visit.

**Fig. 2:**
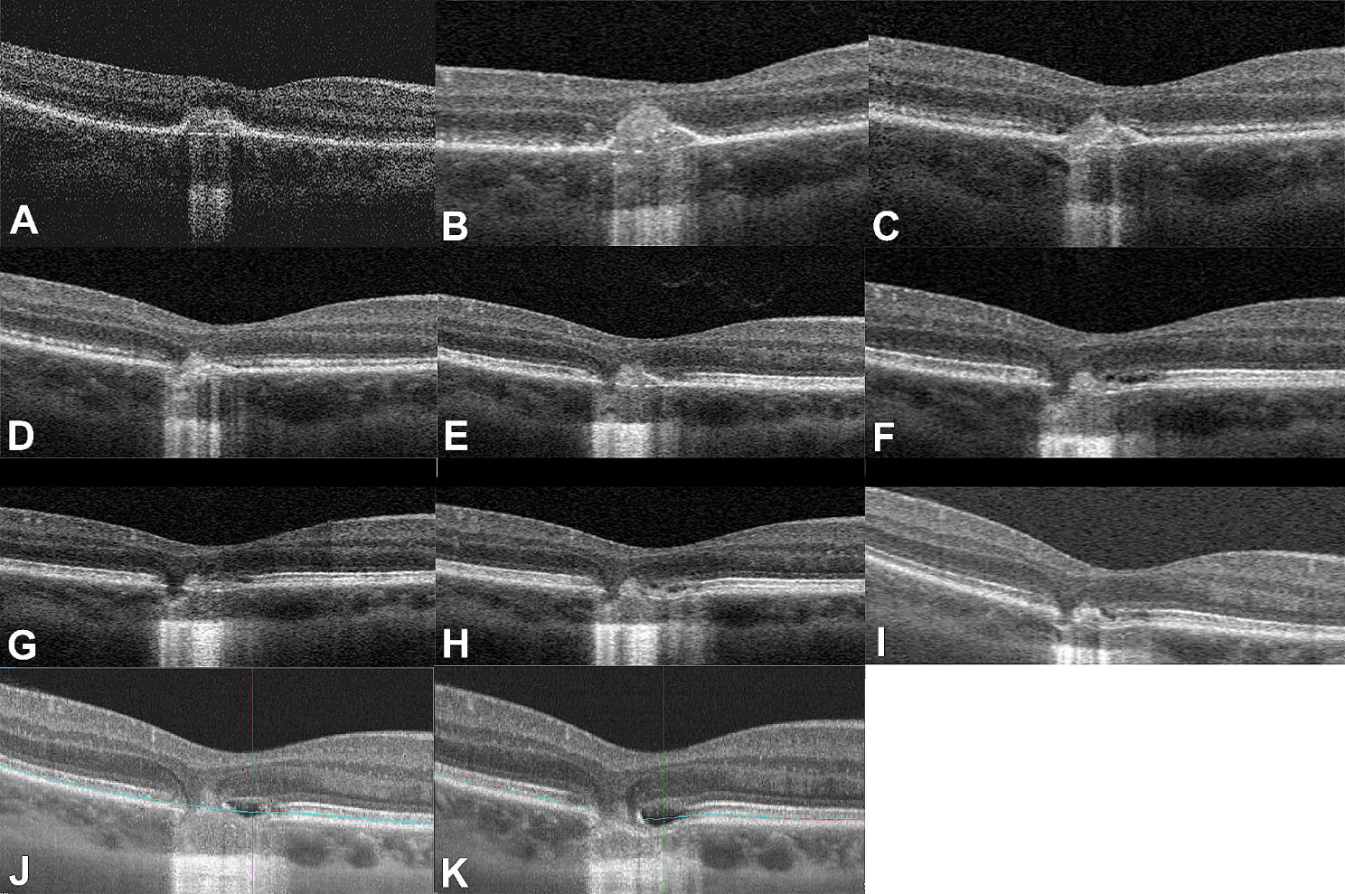
At the first visit, OCT showed a “hump-like” moderately hyper-reflective nodule in the outer retina (**A**). Twenty-three days later, the focal “hump-like” nodule expanded (**B**). The moderately hyper-reflective nodule decreased in height after two (**C**) or three months (**D**). Fifteen months later, OCT showed that defects in the local tissue of the outer retina had formed a V-shaped structure (**E**). Thirty-one (**F**) and forty-three months (**G**) after the initial diagnosis, the structure of the lesion area displayed in the OCT is similar to before. Fifty-six months after the initial diagnosis, OCT showed interruption of the external limiting membrane (ELM), ellipsoid zone (EZ), and retinal pigment epithelium (RPE) bands, forming a more pronounced V- shaped structure (**H**). Seventy months after the initial visit, OCT showed that the outer retina still exhibited a V-shaped structure; However, during this period, the retinal structure remained stable and underwent minimal changes (**I**). Eighty months after the initial visit, the photoreceptors in the lesion area were lost as the cavity was formed locally (**J**). Night-one months after the initial visit, OCT revealed the presence of a focal choroidal excavation (FCE) also with a local cavity (**K**)

At the first visit (Fig. 2A), twenty-three days later (Fig. 2B) and two months later (Fig. 2C), OCT showed that a nodule with moderately hyper-reflectivity breached the retinal pigment epithelium (RPE) and extended toward and replaced the photoreceptor layer until it reached and formed a dome within the outer plexiform layer (OPL). The RPE remnants and Bruch’s membrane at the break gradually dissipated, revealing the choroidal portion of the nodule. The OCT results indicated the presence of a small lesion on the retina, but it caused extensive effects on the retinal area. The bands of the ellipsoid zone (EZ) and external limiting membrane (ELM) were disrupted and discontinuous. Three months (Fig. 2D), fifteen months (Fig. E), thirty-one months (Fig. 2F), forty-three months (Fig. 2G), fifty-six months (Fig. 2H) and seventy months (Fig. 2I) after the initial visit, the nodule regressed from its apex toward the choroidal part, subsequently leading to an incarcerated herniation of the OPL and inner retina, gradually forming a V-shaped appearance through the breach in the RPE and Bruch’s membrane. Eighty months after the initial visit, the photoreceptors in the lesion area were lost as the cavity was formed locally (Fig. 2J). Ninety-one months after the initial visit, OCT revealed the presence of focal choroidal excavation (FCE) also with a local cavity (Fig. 2K).

**Fig. 3:**
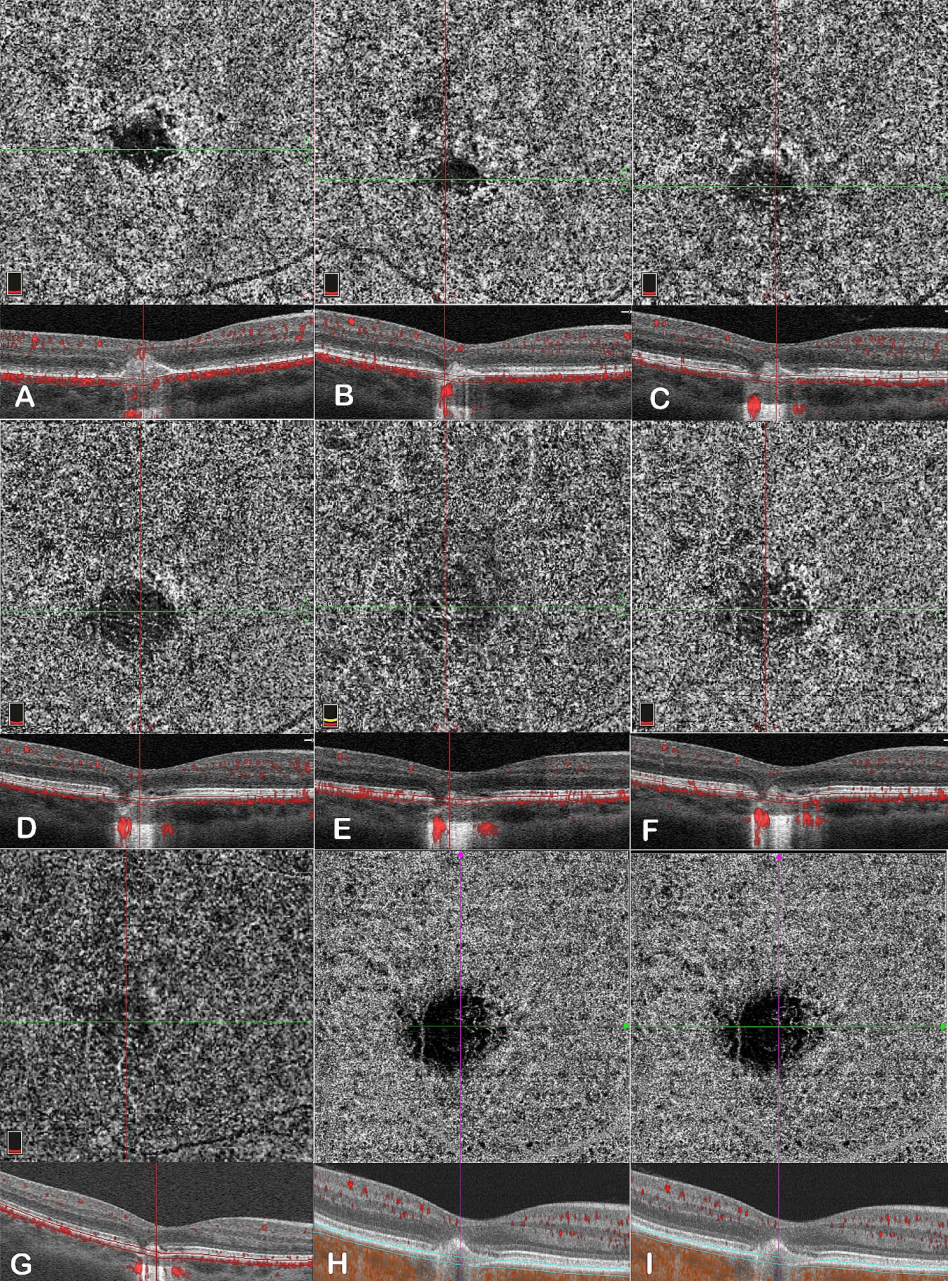
Three weeks (**A**) and three months (**B**) after the initial diagnosis, OCTA showed faintly visible vascular-like structures within the lesion. Fifteen months (**C**), thirty-one months (**D**), forty-three months (**E**), fifty-six months (**F**) and seventy months (**G**) after the initial visit, OCTA showed that there were clearly visible vascular-like structures within the lesion area, and the density of vascular-like structures remained stable during this time. Eighty months (**H**) and ninety-one months (**I**) after the initial visit, OCTA showed a distinct regression of vascular-like structures within the lesion, but it was still faintly visible

Three weeks (Fig. 3A) and three months (Fig. 3B) after the initial diagnosis, OCTA revealed a faintly visible vascular-like network in the lesion. However, fifteen months (Fig. 3C), thirty-one months (Fig. 3D), forty-three months (Fig. 3E), fifty-six months (Fig. 3F) and seventy months (Fig. 3G) after the initial visit, the vascular-like structure in the lesion gradually became clear and remained stable for a long time. Eighty (Fig. 3H) to ninety-one months (Fig. 3I) after the initial diagnosis, OCTA has shown a distinct regression of vascular-like structures within the lesion, but it is still faintly visible. We didn’t find any typical CNV during the whole follow-up period.

**Fig. 4:**
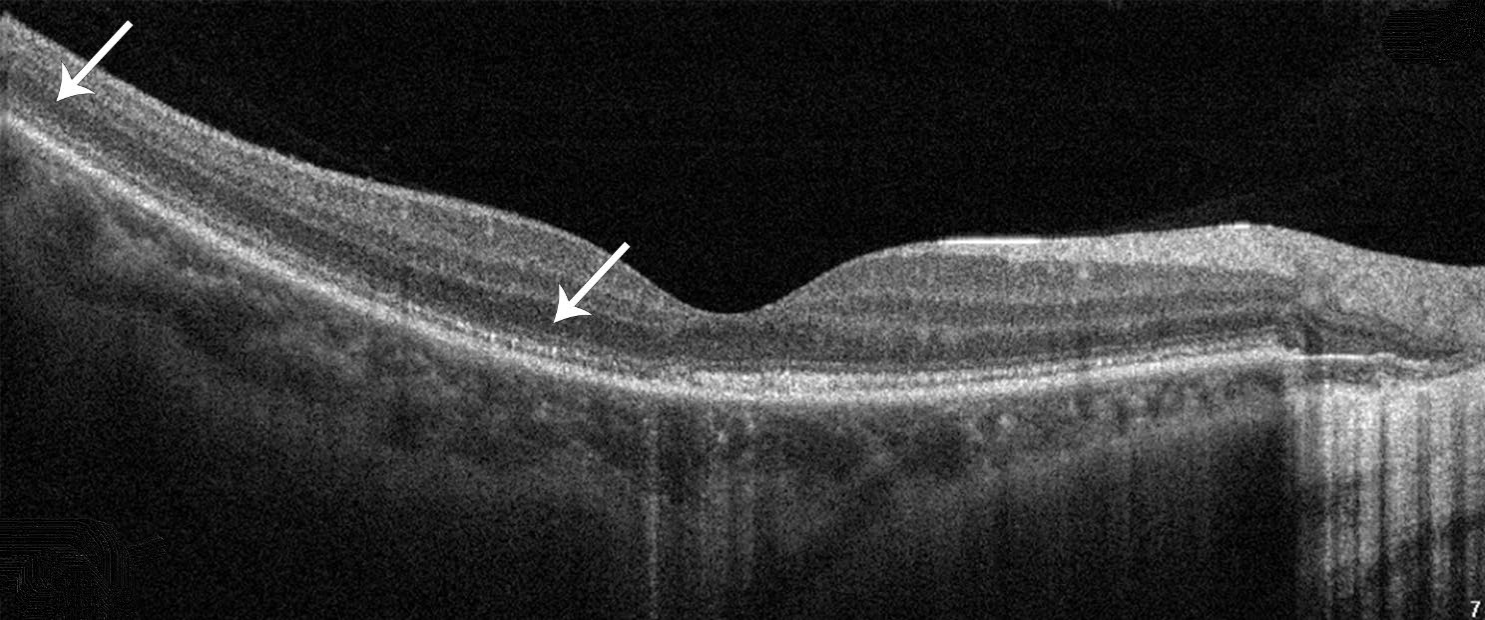
In the first three months after the initial diagnosis, the bands of EZ and ELM on OCT images were disrupted and discontinuous (white arrow)

Within three months of the onset of the disease, FAF showed widespread hyper-autofluorescence extending beyond the macula and optic disc, OCT through the hyper-autofluorescence areas revealed irregularity of the EZ (Fig. 4). However, in subsequent OCT examinations, the outer layers exhibited a normal pattern of hyper-reflectivity.

To evaluate the impact of the lesion on the patient’s visual fields, perimetry and microperimetry were conducted at the first visit and the last follow-up respectively. The initial perimetry revealed central scotomas above and nasal to the central fovea. The mean deviation (MD) value was − 8.06. Ninety-one months later the result of microperimetry elucidated the presence of two yellow dots, which symbolized the reduced sensitivity confined in the lesion area.

## Discussion

Recently, a rare subtype of PIC named SPC with unique features in multimodal imaging has been reported [9]. The lesion of SPC is solitary, small and located near the foveal area. SPC may recur at the same location and lead to FCE or CNV, which can be detected on OCTA noninvasively. Though the SPC lesion may resolve spontaneously, recurrence and complications warrant attention for the management. Gan et al. [9] reported a low incidence of SPC, with the lesion size typically ranging from 0.12 mm² to 2.46 mm². Throughout follow-up, The SPC lesion tends to remain solitary and localized near the macular fovea, without progressing to involve additional lesions. A subset of patients may experience recurring inflammation at the same site rather than developing inflammatory lesions elsewhere. The isolated punctate lesion often persists, giving rise to the formation of a retinal cystic cavity, FCE, or CNV.

According to a previous study, PIC lesions can be categorized into five stages: choroidal infiltration, formation of sub-RPE nodules, and then chorioretinal nodules, regression, and retinal herniation, as demonstrated in a 20-month follow-up study conducted by Zhang et al. [10] in 2010. Our study is dedicated to observing a long prognosis of SPC. After the initial visit, OCT revealed the presence of a “hump-like” moderately hyper-reflective nodule in the outer layer of the retina, which was consistent with the third stage of PIC. A V-shaped hernia appeared 15 months later. Subsequently, there was a gradual loss of photoreceptors in the lesion area, resulting in the formation of localized cavities. This overall pattern matches the changes in the latter three stages of PIC discovered by Zhang and associates [10]. Even after 91 months of follow-up, the fundus still maintained a solitary lesion.

Concerning the treatment of individuals with this condition, Gan et al. reported two cases of CNV successfully managed with anti-VEGF therapy [9]. In our case, we observed a vascular-like structure that did not convert into a typical CNV during the whole follow-up period. OCTA from 2016 to 2023 showed that the vascular-like structure within the lesion remained stable and largely spontaneously resolved without the need for anti-VEGF therapy. This observation suggested that vessels induced by our case with SPC could be characterized as quiescent CNV [2, 11]. The term “quiescent CNV” was initially coined by Querques et al. [12–17]. The diagnostic criterion for quiescent CNV is the absence of intraretinal or subretinal exudation on repeated structural SD-OCT examinations over at least 6 months and late-phase ill-defined hyperfluorescent lesions without late-phase leakage or pooling of dye on FFA [12]. In our follow-up observations, the patient met the criteria of no intraretinal or subretinal exudation on repeated structural SD-OCT examinations over at least 6 months. Although there was staining in late stage FFA, it was primarily caused by the lesion itself rather than the CNV. If CNV had been the cause of the staining, there would generally be leakage of fluorescein, and as time progressed, the extent of the lesion would have significantly enlarged [18]. However, the patient’s FFA showed only faint hyperfluorescence at the site of the lesion, with no obvious fluorescein leakage detected even in the late phase. Regarding the treatment of typical CNV, the most widely used approach is anti-VEGF therapy. However, the need for anti-VEGF treatment for a patient depends on several factors. On one hand, anti-VEGF therapy can be expensive, have side effects, and carry some risks. On the other hand, the lesion is located near the fovea. Once the lesion spreads, it can greatly affect vision. Therefore, a careful evaluation is necessary to weigh the risks and benefits. Considering that anti-VEGF treatment has shown significant efficacy in treating active CNV lesions, its effectiveness on quiescent CNV is less clear. In this case, we ultimately decided not to pursue anti-VEGF treatment and instead opted for close monitoring of the patient’s condition.

Although the fundus photography indicated the presence of a small lesion on the macula (Fig. 1A) FAF revealed extensive effects on the posterior retina with abnormal hyper-autofluorescence (Fig. 1C). The abnormal hyper-autofluorescence (Fig. 1C) corresponded to the disruption of the bands of EZ and ELM (Fig. 4). This may represent an early sign of outer retinal inflammation [19]. To avoid pupil dilation, we did not re-examine FAF. However, microperimetry was able to reflect some recovery. Microperimetry found no issues in other areas, which also demonstrates that the lesion corresponding to the yellow dot did not progress further, compensating for the limitation of not conducting FAF. In the initial stages of the lesion, the patient’s visual field showed a central scotoma with a wider range. During the last visit, microperimetry revealed that the lesion was confined to a smaller area, indicated by yellow numbers, which suggested functional repair. During the initial follow-up, OCT showed gradual restoration of the outer retinal structure, confirming the stability of the patient’s condition. This was another reason for not performing FAF again.

As a subtype of white dot syndrome (WDS), SPC, PIC and Multiple evanescent white dot syndrome (MEWDS) share some common features. To differentiate SPC from other diseases, we need to compare SPC with PIC and MEWDS separately. The main distinguishing factor between SPC and PIC is the number of lesions. SPC typically presents with a solitary lesion, while PIC may have two or more lesions. Patients with SPC are less likely to develop CNV than those with PIC, and the probability of secondary CNV in SPC is only 16% [9], compared to nearly 50% in PIC [20]. MEWDS has unique characteristics, such as a halo-like hyper-fluorescence on FFA and fluorescein staining around the optic disc in the late phase of FFA [21, 22], which were not seen in our case.

## Conclusions

SPC represents a recently identified entity characterized by a small, solitary lesion located in proximity to foveal region. SPC can affect young myopic females and exhibits a distinctive appearance on OCT. While we are not the first to report on this specific case of SPC, our significant advantage lies in the extensive duration of follow-up. Over a follow-up period of nearly ninety-one months, the lesion near the fovea has remained isolated, further supporting the possibility that SPC may be a distinct subtype of PIC. In cases where suspicious CNV occurs in SPC patients, aggressive anti-VEGF treatment may not be necessary, but close monitoring is still required.

## Data Availability

The original contributions presented in the study are included in the article/supplementary material, further inquiries can be directed to the corresponding author.
